# Alcoholic Fractions F5 and F6 from *Withania somnifera* Leaves Show a Potent Antileishmanial and Immunomodulatory Activities to Control Experimental Visceral Leishmaniasis

**DOI:** 10.3389/fmed.2017.00055

**Published:** 2017-05-12

**Authors:** Sambamurthy Chandrasekaran, Jalaja Veronica, Shyam Sundar, Radheshyam Maurya

**Affiliations:** ^1^Department of Animal Biology, University of Hyderabad, Hyderabad, India; ^2^Infectious Disease Research Laboratory, Institute of Medical Sciences, Banaras Hindu University, Varanasi, India

**Keywords:** visceral leishmaniasis, peritoneal mouse macrophages, *Withania somnifera*, withaferin-A, IL-10

## Abstract

Visceral leishmaniasis (VL) causes fatal life-threatening disease, if left untreated. The current drugs have various limitations; hence, natural products from medicinal plants are being focused in search of new drugs to treat leishmaniasis. The aim of the present study was to evaluate the antileishmanial and immunomodulatory activities of F5 and F6 alcoholic fractions from *Withania somnifera* leaves and purified withaferin-A in *Leishmania donovani*-infected peritoneal macrophages and BALB/c mice. We observed that F5 (15 µg/mL), F6 (10 µg/mL), and withaferin-A (1.5 µM) reduce amastigote count in peritoneal macrophages and induce reactive oxygen species and significant decrease in IL-10 mRNA expression compared to control upon treatment. Subsequently, *in vivo* study mice were treated with F5 (25 and 50 mg/kg b.wt.), F6 (25 and 50 mg/kg b.wt.) orally, and withaferin-A (2 mg/kg b.wt.) intraperitoneally for 10 consecutive days and a drastic reduction in parasite burden in both spleen and liver were observed. The treatment resulted in the reduction in IL-10, IL-4, and TGF-β mRNA expression and a significant increase in IFN-γ/IL-10 expression ratio in the treated group compared to control. The humoral response of these alcoholic fractions and withaferin-A shows increased IgG2a levels when compared with IgG1 in treated mice. Taken together, our result concludes that withanolides in alcoholic fractions demonstrate a potent antileishmanial and immunomodulatory activities in experimental VL.

## Introduction

Visceral leishmaniasis (VL) is one of the most severe forms of leishmaniasis, which affects the visceral organs *viz*. spleen and liver. It poses a serious public health problem and is responsible for most of the *Leishmania*-associated deaths all over the world (1). In over 98 countries, more than 350 million people are at risk of leishmaniasis (2, 3) and 200,000–400,000 new cases of VL occur each year globally, of which one-tenth are fatal (2, 4). Currently, adopted treatment strategies are inadequate due to high cost and/or adverse events of the antileishmanial drugs. Together this, growing drug resistance is further affecting the efficacy (5). The first line of treatment constitutes of pentavalent antimony compounds, in which the resistance is widespread in Bihar, India (6). Currently used antileishmanial drugs are liposomal amphotericin-B and an oral drug miltefosine; however, both have several drawbacks (7). In VL, resolution and resistance to the disease depend on the balance of Th1/Th2 dichotomy, and induction of Th2 confers its susceptibility (8, 9). Major cytokines produced by Th1 cells, include IFN-γ, IL-2, IL-12, and TNF-α, are crucial for disease resistance whereas, IL-4, IL-10, and TGF-β produced by Th2 cells promote disease progression (9). Activated macrophages play a critical role in innate and adaptive immune response through secretion of toxic molecules such as reactive oxygen species (ROS) and nitric oxide (NO), which show cytotoxic effects against several pathogens (10).

Medicinal plants having anti-infectious properties have been used as a natural source to identify new antiparasitic compound and source of immunomodulators (11), and their use has been increasing in the developed countries. *Withania somnifera* is a green shrub belonging to the family Solanaceae, which is commonly known as Ashwagandha, or winter cherry has been used for the treatment of various diseases in the Indian traditional medicine for over 3,000 years (12, 13). Moreover, *W. somnifera* was found to exert anti-inflammatory (14), anticancer (15), antioxidant (16), and immunomodulatory (17, 18) activities. *Withania somnifera* contains biologically active constituents in their leaves and roots known as withanolides, which belong to highly oxygenated steroidal lactones group derived from a C28 ergostane skeleton (19). Withaferin-A and withanolide-E exhibited a specific immunosuppressive effect on human B and T lymphocytes and mice thymocytes. Withanolide-E had an effect on T lymphocytes, whereas withaferin-A affected both B and T lymphocytes (20). The methanolic extracts from *W. somnifera* were reported to possess antileishmanial activity *in vitro* (21, 22). A recent study also revealed that the antileishmanial activity of the herbal drug from *W. somnifera* is exhibited through blocking of the protein kinase C signaling pathway (23). The treatment of infected mice with a combination of *Asparagus racemosus* and *W. somnifera* extracts not only resulted in the successful reduction in parasite level but also generated protective Th1-type immune responses with normalization of biochemical and hematological tests, suggesting their role as an important antileishmanial agent (24).

Withaferin-A has been identified as one of the prominent withanolides from *W. somnifera* and has shown antileishmanial activities through the inhibition of protein kinase activity of DNA topoisomerase-I, which induces apoptosis through apoptotic topoisomerase I–DNA complex (22). Earlier studies from our group demonstrated that the alcoholic fraction of F5 and F6 from *W. somnifera* leaves showed potent antileishmanial activity against promastigotes stage of the parasites. Among the seven fractions of alcoholic extract, fractions F5 and F6 were further subjected to high-performance liquid chromatography and liquid chromatography-mass spectrometry to confirm the presence of various withanolides including withaferin-A (25). Furthermore, molecular docking and enzyme inhibition studies revealed that withaferin-A, an abundant withanolide present in the F5 and F6 fractions inhibit pteridine reductase-1 enzyme in the Pteridine salvage pathway of the parasite (26), exclusively present in trypanosomatids.

However, its efficacy against chronic experimental VL has not yet been demonstrated. In the present study, we have evaluated the F5 and F6 bioactive fractions from *W. somnifera* leaves and withaferin-A, an abundant withanolide present in leaves, for their antileishmanial and immunomodulatory activities in *ex vivo* mouse peritoneal macrophages and *in vivo* BALB/c model.

## Materials and Methods

### Parasites

*Leishmania donovani* strain MHOM/IN/80/DD8 was obtained from the ATCC, USA, and cultured as promastigotes at 25 ± 1°C in M199 media supplemented with 15% fetal bovine serum (FBS) and 1% penicillin–streptomycin antibiotic mixture. BALB/c mice of 4 weeks old reared in institute facilities were used for parasite maintenance.

### Animals

The 6- to 8-week-old female BALB/c mice were obtained from National Centre for Laboratory Animal Sciences (NCLAS), Hyderabad, India. The animals were kept and fed with water and mouse feed *ad libitum* in the Animal House facility of University of Hyderabad. All animal experiments were approved by the Institutional Animal Ethics Committee, University of Hyderabad (UH/IAEC/RSM/2014-I/29a).

### Drugs

F5 and F6 fractions were isolated from *W. somnifera* leaves as described elsewhere (25). Briefly, the alcoholic fractions of F5 and F6 were lyophilized in SpeedVac Concentrators (Thermo Scientific™) and redissolved the lyophilized powder at the concentration of 1 mg/mL in 100% dimethyl sulfoxide (DMSO). The working dilution of both fractions was prepared in complete media, and the final concentration of DMSO was kept at 0.1% in all experiments. Withaferin-A was purchased from Sigma-Aldrich Co., USA, and dissolved in methanol at 1 mg/mL concentration. The drug was further diluted with media prior to the experiment.

### *Ex Vivo* Study

#### Isolation of Peritoneal Mouse Macrophages (PMM)

Peritoneal macrophages were isolated from BALBc mice as described by elsewhere (27, 28). Briefly, mice were injected 4% sterile thioglycollate broth intraperitoneally and kept them for 48 h. After incubation, mice were again injected with 2 mL of ice-cold RPMI 1640 media with 10% FBS. The peritoneal cavity walls were massaged to allow the detachment of the adherent cells and aspirated the cells. The cells were washed and resuspended in RPMI 1640 media. Finally, the cells were plated in 100 mm culture dish and incubated at 37°C and 5% CO_2_ for 24 h for macrophage adhering and allowing them to rest to revert to normal state if any of the macrophage is stimulated due to the thioglycollate broth.

#### Cytotoxicity Assay

Toxicity of the F5, F6, and withaferin-A on PMM was evaluated using MTT (3-(4, 5-dimethylthiazol-2-yl)-2,5-diphenyltetrazolium bromide) assay. Briefly, 1 × 10^5^ cells were seeded in 200 µL volume in 96-well plate. F5, F6, and withaferin-A were used at different concentrations, and the plate was incubated at 37°C with 5% CO_2_ for 72 h. Furthermore, the plate was spun at 1,000 *g* for 5 min; the fresh media were carefully changed and incubated at 37°C with 5% CO_2_ for 4 h with 20 µL of 5 mg/mL MTT solution. Next, the plate was spun at 1,000 *g* for 5 min and the media was discarded. Finally, 100 µL of DMSO was added to dissolve the purple formazan crystals, and absorbance was taken at 540 nm.

#### Macrophage Infection

*Ex vivo* assessment of antileishmanial activity was done with peritoneal macrophage. Cells (2 × 10^5^) were seeded on coverslips, and after 24 h of incubation, nonadherent cells were washed and parasites were added at 1:10 ratio. The infection was allowed for 16 h at 37°C and nonphagocytized parasites were washed three times with serum-free RPMI 1640 medium. F5 (15 µg/mL), F6 (10 µg/mL), and withaferin-A were added at different concentrations (0.5, 1.0, and 1.5 µM) and incubated for additional 72 h. Coverslips were removed, fixed with methanol, and stained with Giemsa stain. Number of amastigotes were counted per 100 macrophages and compared with untreated macrophages.

#### Estimation of ROS

Intracellular ROS such as HO^⋅^, HOO^⋅^, and $O_{2}^{-}$ radicals are estimated using H_2_DCFDA dye (29). The dye is a nonpolar molecule, which freely enters the cell where the acetate moiety of the molecule is cleaved by the cellular esterase to release the nonfluorescent dicholorofluorescein. The fluorescent intensity is directly proportional to the amount of ROS produced in the cell. Briefly, PMM were treated with F5 (15 µg/mL), F6 (10 µg/mL), withaferin-A (1.5 µM), miltefosine (3.2 µM), and LPS (100 ng/mL) and incubated at 37°C for 6 h. After incubation, cells were washed and incubated with H_2_DCFDA at a final concentration of 10 µM for 15 min at room temperature. Finally, cells were washed once and acquired on BD™ LSR Fortessa flow cytometer. The production of ROS was analyzed by measuring their mean fluorescence intensities using FACS Diva software.

#### Estimation of IFN-γ and TNF-α

To evaluate the immune alterations, we have estimated the concentration of cytokines in culture supernatants of peritoneal macrophages. Briefly, after various treatments, the culture supernatants were used to measure the amount of IFN-γ and TNF-α using mouse Th1/Th2 cytometric bead array (BD Biosciences) in BD™ LSR Fortessa flow cytometer. The concentrations of cytokines were measured in terms of pictograms per milliliter by comparing with the standards of the respective cytokines.

### *In Vivo* Study

#### Experimental Design

Inbred BALB/c mice were infected with 1 × 10^8^ stationary phase promastigotes of *L. donovani* through the tail vein injection. After 30 days of infection, mice were treated with F5 (25 and 50 mg/kg b.wt./day for 10 days, orally), fraction F6 (25 and 50 mg/kg b.wt./day for 10 days, orally), withaferin-A (2 mg/kg b.wt./day for 10 days, intraperitoneally) and the concentration was decided from the previous study (30). Mice treated with miltefosine (5 mg/kg b.wt./day for 10 days, orally) served as positive control. Infected mice but not treated with any drug served as infected controls. Uninfected control mice were given PBS. Each group consisted of five mice.

#### Assessment of Infection

All mice from each group were sacrificed 45-day postinfection/posttreatment. Splenic and hepatic parasite burdens were determined from Giemsa-stained multiple impression smears, expressed as *Leishman-Donovan units* as per the method of Ref. (31). The parasite reduction in treatment groups was represented as percentages to assess the degree of cure.

#### RT-qPCR Analysis of Cytokine Genes

Total RNA was extracted from 5 × 10^6^ peritoneal macrophages and 25 mg of spleen tissue using a Nucleospin RNA extraction kit (Machery-Nagel) following the instructions of the manufacturer. The 900 ng of total RNA was then reverse transcribed into cDNA using a First Strand cDNA synthesis kit (Takara). The cDNA samples were stored at −80°C until use. The resulting cDNA was then used for real-time PCR for iNOS and cytokines (IL-12p40, IL-10, IFN-γ, IL-4, and TGF-β) using an ABI 7500 real-time PCR system (Applied Biosystems, UK) with the DNA-binding SYBR green dye. Glyceraldehyde-3-phosphate dehydrogenase (GAPDH) was used as a reference. The forward and reverse specific primer sequences are given in Table 1. The detection of the dequenched probe, calculation of threshold cycles (C_t_ values), and the data analysis were performed using the Sequence Detector software. Relative changes in iNOS and cytokine (IL-12p40, IL-10, IFN-γ, IL-4, and TGF-β) mRNA expression were compared with unstimulated control, normalized to GAPDH, and were quantified by the Δ2^−ddCt^ method. Thus, all the values for experimental samples were expressed as fold differences between the sample mRNA and the calibrator (GAPDH) mRNA. The data are represented as mean ± SD of data from three independent experiments that yielded similar results.

**Table 1:** **Forward and reverse primer sequences specific for RT-qPCR**.

S. no.	Name	Sequence
1	iNOS	Forward 5′-CAGCTGGGCTGTACAAACCTT-3′
		Reverse 5′-CATTGGAAGTGAAGCGTTTCG-3′
2	IL-10	Forward 5′-GGTTGCCAAGCCTTATCGGA-3′
		Reverse 5′-ACCTGCTCCACTGCCTTGCT-3′
3	IL-12p40	Forward 5′-GGAAGCACGGCAGCAGAATA-3′
		Reverse 5′-AACTTGAGGGAGAAGTAGGAATCG-3′
4	IFN-γ	Forward 5′-TCAAGTGGCATAGATGTGGAAGAA-3′
		Reverse 5′-TGGCTCTGCAGGATTTTCATG-3′
5	IL-4	Forward 5′-ACAGGAGAAGGGACGCCAT-3′
		Reverse 5′-GAAGCCCTACAGACGAGCTCA-3′
6	TGF-β	Forward 5′-TGACGTCACTGGAGTTGTACGG-3′
		Reverse 5′-GGTTCATGTCATGGATGGTGC-3′
7	GAPDH	Forward 5′-CAAGGCTGTGGGCAAGGTCA-3′
		Reverse 5′-AGGTGGAAGAGTGGGAGTTGCTG-3′

#### Estimation of Th1/Th2-Specific IgG from Sera

The levels of IgG1 and IgG2a antibodies in sera samples of mice of different experimental groups were measured. Briefly, 96-well ELISA plates were coated with soluble *Leishmania* antigen (0.25 µg/100μl/well) in coating buffer (0.1 M sodium bicarbonate pH 9.6) and incubated at 4°C for overnight. Plate was washed with PBS-Tween 20 and blocked with 1.5% bovine serum albumin at room temperature for 1 h. Sera was used at a dilution of 1/100 for IgG1 and IgG2a and kept for 2 h at room temperature. HRP-conjugated rat anti-mouse IgG1 and IgG2a (Abcam) were added for 1 h at room temperature at 1/10,000 dilutions. Finally, the substrate TMB/H_2_O_2_ was added, kept in dark for 30 min, and 50 µL stop solution was added, and the plate was read at 450 nm.

#### Histopathological Examination

Hepatic tissue from control and treated mice was isolated and fixed in 10% formalin in PBS. Wax blocks were made, and sections were stained with hematoxylin/eosin stain for histological studies. The number of granulomas per 50 fields was counted using a light microscope (Leica).

## Results

### Cytotoxicity Assay

**Figure 1:**
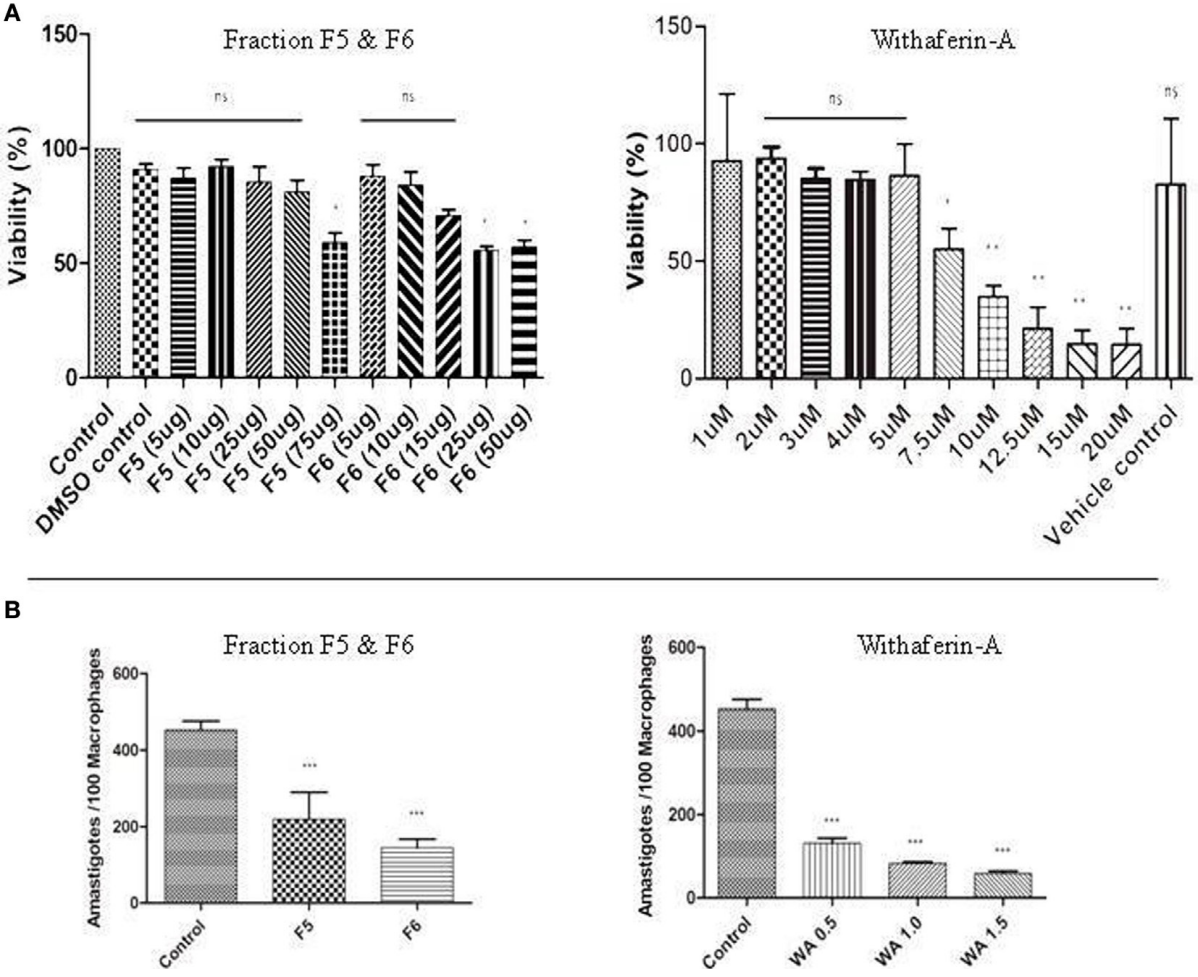
**(A)** Cytotoxic assay and parasite burden. MTT assay with fraction F5, F6, and withaferin-A. The results are represented as percentage of viability compared to control. **(B)** Antileishmanial activity of F5, F6, and withaferin-A on intracellular amastigotes using *ex vivo* peritoneal mouse macrophages (PMM) was counted using Giemsa staining. The graph shows the number of amastigote/100 macrophages treated with F5 (15 µg/mL), F6 (10 µg/mL), and withaferin-A (0.5 µM, 1.0 µM, and 1.5 µM). The values are from three independent experiments, and significance values were calculated using unpaired Student’s *t*-test (*P < 0.05, **P < 0.01, and ***P < 0.001).

The toxicity assay on PMM was determined by MTT assay. Briefly, we treated PMM with different concentrations of active fractions F5, F6, and withaferin-A for 72 h. The results indicated that treatment with F5 decreased the cell survivability of PMM by just 40% at the highest concentration of 75 µg/mL, whereas F6 induced the similar effect at 50 µg/mL. In case of withaferin-A, there was decrease in survivability by 40% at 7.5 µM concentration. Furthermore, increase in withaferin-A concentration pronounced more death of PMM. Hence, the concentration of F5 up to 25 µg/mL, F6 up to 25 µg/mL, and withaferin-A up to 3 µM were further considered determining its antileishmanial activity in PMM (Figure 1A).

### Withanolides Suppress Intracellular Parasite Burden

The efficacy of antileishmanial properties of F5, F6, and withaferin-A was determined by counting intracellular parasite load in the treated PMM after 72 h using Giemsa staining. We treated the infected PMM with different concentrations of F5 and F6 fractions and observed that F5 at 15 µg/mL and F6 at 10 µg/mL could reduce the parasite load by ~50% (*P* < 0.001) and ~60% (*P* < 0.001), respectively, compared to infected control. Furthermore, in the case of withaferin-A, we observed a dose-dependent decrease in the parasite number with increase in its concentration (0.5, 1.0, and 1.5 µM). The decrease was ~80% (*P* < 0.001) with 1.5 µM concentration, and we used this concentration for further studies as it showed minimum cytotoxicity against the naive PMM. These results indicate that F5, F6, and withaferin-A exhibit antileishmanial activity on the intracellular stage of the parasite with the decrease in parasite load being more than 50% in all cases (Figure 1B).

The safety index (SI) of the withaferin-A was calculated by considering the highest concentration of withaferin-A on the macrophages and concentration on the infected macrophages, which ranges from 5 to 15. The SI value less than 2 indicates general toxicity of the pure compound on normal cells (32, 33). Hence, in this study, we have considered the lower range of SI value.

### Withanolides Induces ROS-Mediated Parasite Killing

**Figure 2:**
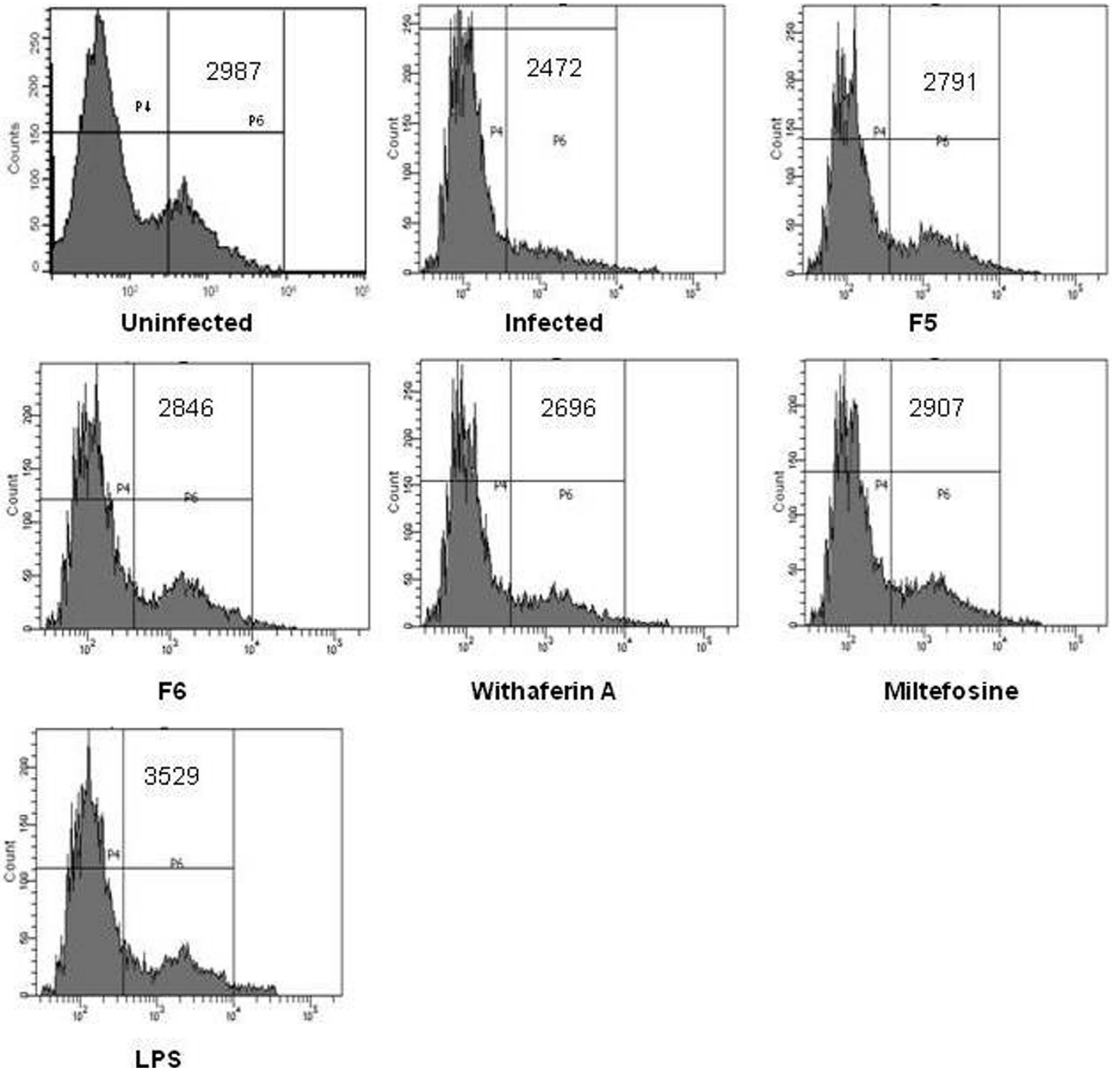
**Reactive oxygen species (ROS) production from peritoneal mouse macrophages (PMM) was estimated using a fluorescence dye H_2_DCFDA**. The graph shows the mean fluorescence intensity (MFI) values of uninfected, infected, F5 (15 µg/mL), F6 (10 µg/mL), withaferin-A (1.5 µM), miltefosine (3.2 µM), and LPS (100 ng/mL) treated PMM.

Reactive oxygen species is one of the key mediators of microbicidal molecule used by the macrophages for their defense against the pathogens (34). In our study, we found no significant increase in NO production in treated macrophages. This prompted us to examine the ROS levels in the macrophages. We treated the macrophages with F5, F6, and withaferin-A for 6 h, and ROS production was measured using H_2_DCFDA dye. There was a significant increase in the mean fluorescence intensity when treated with F5 (2,791), F6 (2,846), and withaferin-A (2,696) when compared with infected macrophages (2,472). Miltefosine, used as a positive control for treating *Leishmania* infections, was also able to induce ROS levels (2,907) in PMM. LPS being a bacterial cell wall component could induce ROS in huge amounts. So, our results indicate that F5, F6, and withaferin-A might demonstrate the antileishmanial activity by inducing the ROS in PMM (Figure 2).

### Withanolides Suppresses IL-10-Mediated Macrophage Activity

**Figure 3:**
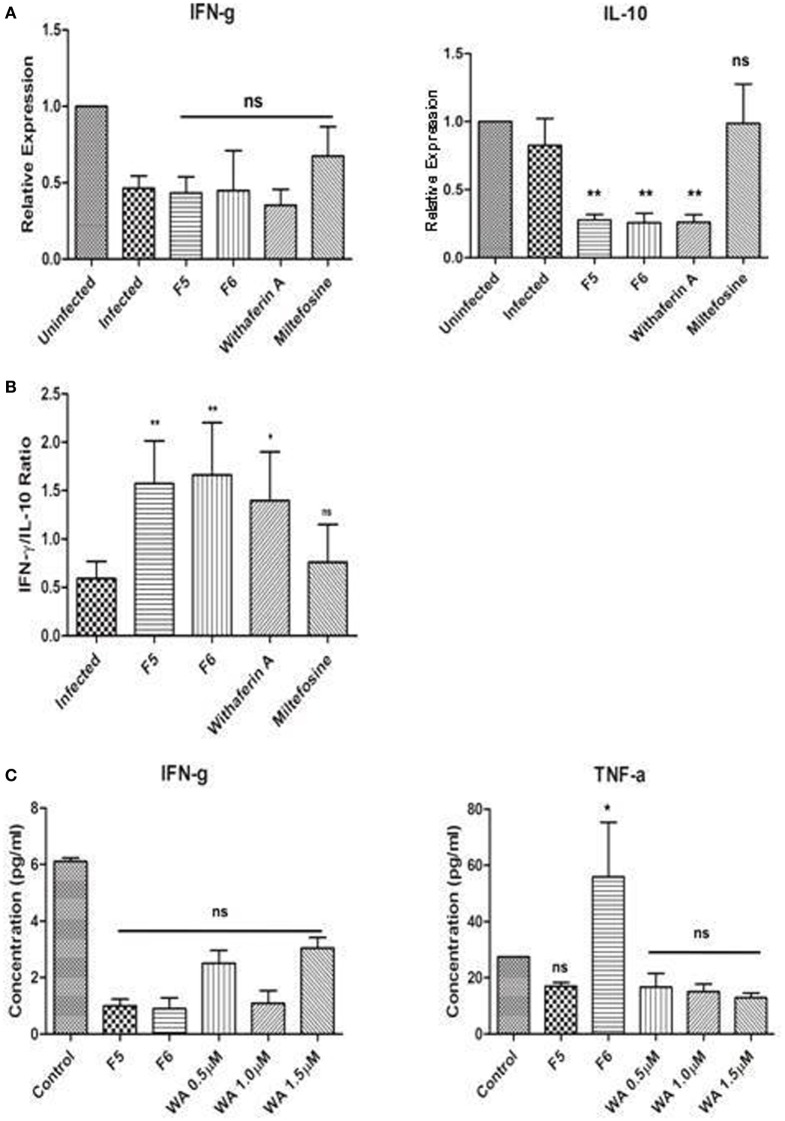
**Immunomodulatory effect of the withanolides in infected control and treated peritoneal mouse macrophages (PMM) *ex vivo***. **(A)** RT-qPCR analysis of IFN-γ and IL-10 cytokines. **(B)** IFN-γ/IL-10 ratio representing the parasite clearance from PMM. **(C)** IFN-γ and TNF-α protein levels in PMM culture supernatants were assessed using CBA analysis. Unpaired Student’s *t*-test was performed (*P < 0.05, **P < 0.01, and ***P < 0.001).

The outcome of the *Leishmania* infection depends on the balance between Th1 and Th2 cytokines, of which the Th1 favors the parasite clearance from the macrophages. To confirm this, we measured the mRNA expression of IFN-γ (Th1) and IL-10 (Th2) cytokines using RT-qPCR in the infected and treated PMM. The expression of IL-10 being crucial for the parasite persistence in the macrophages was significantly downregulated in F5-, F6-, and withaferin-A-treated PMM when compared with infected control (Figure 3A). We did not observe any alteration in IFN-γ expression among all the treated PMM compared to infected control. When the ratio of IFN-γ/IL-10 was taken into account, we observed that the increase in ratio directly correlates with the reduction in the parasite burden in treated macrophages when compared with infected control. The IFN-γ/IL-10 ratio is a good indicator of infection resolution both *in vitro* and *in vivo* (Figure 3B). Simultaneously, we also measured the cytokine levels (IFN-γ and TNF-α) in culture supernatants of PMM using CBA kit. In corroboration with our RT-qPCR results, we did not find any significant increase in IFN-γ levels in the treated PMM compared to infected control. While TNF-α levels were significantly induced in F6-treated PMM when compared with infected PMM. However, other treatments did not induce any TNF-α (Figure 3C). The results indicate that clearance of the intracellular parasite might depend on decreased IL-10 mRNA suppression in macrophages.

### Withanolides Treatment Reduces Parasite Load in Visceral Organs of Mice

**Figure 4:**
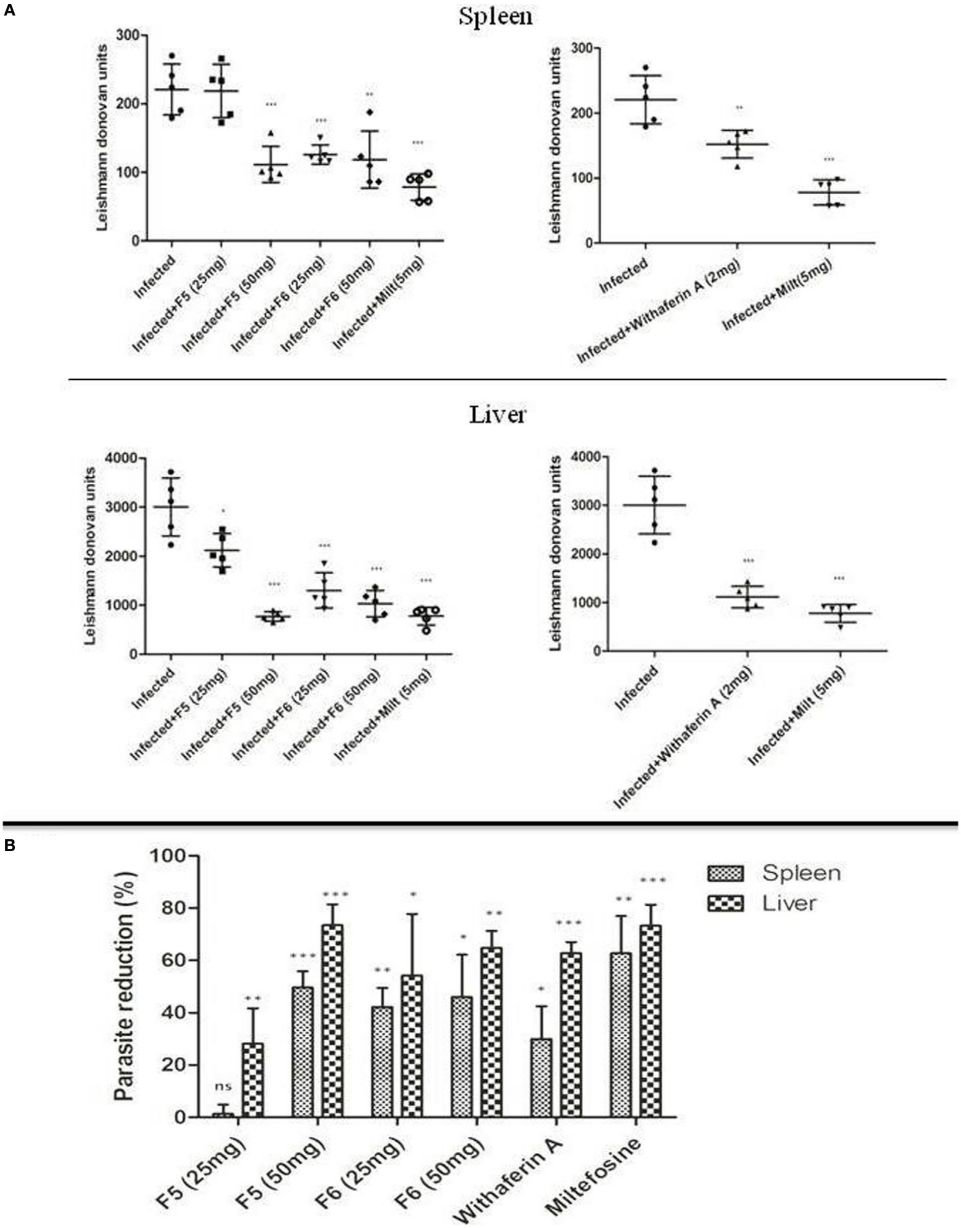
**Antileishmanial effect of withanolides in BALB/c model of visceral leishmaniasis**. **(A)** *Leishman-Donovan* units (LDU) in spleen and liver of infected control and treated mice groups after 45-day postinfection/posttreatment. **(B)** The bar graph represents the percentage of parasite reduction compared to infected control. The two independent experiments were done with five mice per group, and the significance between infected control and treated groups was calculated using one-way ANOVA with Dunnett’s posttest (*P < 0.05, **P < 0.01, and ***P < 0.001).

The efficacy of F5, F6, and withaferin-A was evaluated in chronic experimental VL by estimating the splenic and hepatic parasite burden at 45-day postinfection/posttreatment period. Lower dose treatment with F5 (25 mg/kg b.wt.) did not alter the splenic parasite load, but there was a significant decrease in hepatic parasite burden (*P* < 0.05) at the same dose. On the other hand, treatment with F5 (50 mg/kg b.wt.) fraction showed significant reduction in the parasite load in both spleen (*P* < 0.001) and liver (*P* < 0.001). In contrary, both F6 concentrations, i.e., 25 and 50 mg/kg b.wt. showed a significant reduction in splenic (*P* < 0.001) and hepatic (*P* < 0.001) parasite burden. Treatment with pure compound, withaferin-A (2 mg/kg b.wt.) showed a drastic decrease in the hepatic parasite burden (*P* < 0.001) compared to that of splenic parasite burden (*P* < 0.01). Miltefosine was used as a positive control to demonstrate the efficacy of the treatment (Figure 4A). Furthermore, we calculated the percentage of reduction in splenic and hepatic parasite burdens of treated mice compared to untreated mice to show the effectiveness of treatment in clearing the parasite. The reduction in parasites in spleen and liver was 2 and 30% with F5 (25 mg/kg b.wt.), 50 and 75% with F5 (50 mg/kg b.wt.), 44 and 55% with F6 (25 mg/kg b.wt.), 50 and 65% with F6 (50 mg/kg b.wt.), and 32 and 62% with withaferin-A (2 mg/kg b.wt.), respectively (Figure 4B). The results in part indicate that the treatments with F5, F6, and withaferin-A induce parasite clearance in both the spleen and liver.

### Withanolides Promotes Th1-Dependent Parasite Clearance in VL Spleen

**Figure 5:**
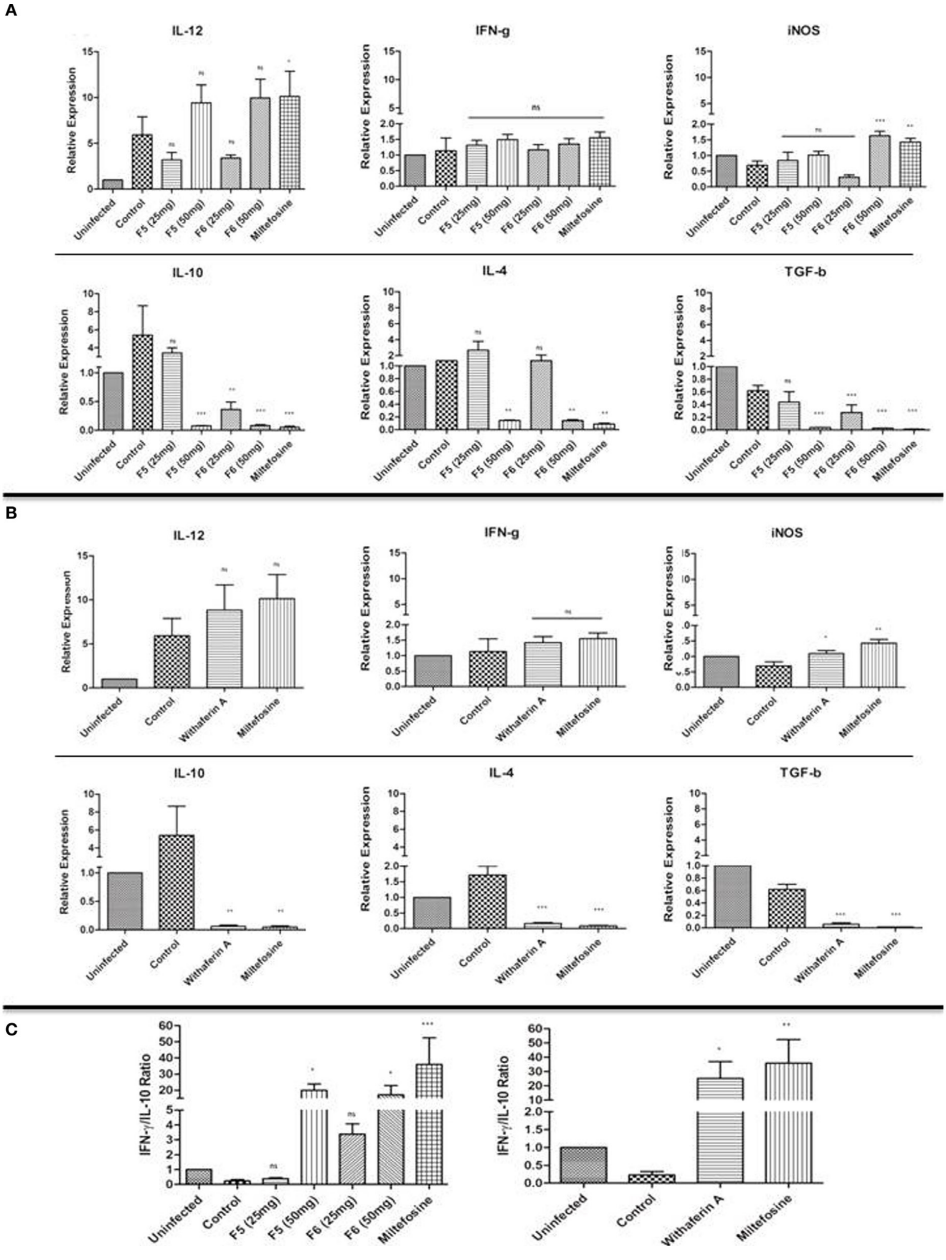
**Immunomodulatory effects of F5, F6, and withaferin-A in BALB/C mice spleen tissue using RT-qPCR analysis**. **(A)** mRNA expression analysis of Th1, Th2 cytokines, and iNOS gene expression in F5- and F6-treated mice after 45-day postinfection/posttreatment. **(B)** mRNA expression analysis of Th1, Th2 cytokines, and iNOS gene expression in withaferin-A-treated mice. **(C)** The graph depicts the IFN-γ/IL-10 ratio in F5-, F6-, and withaferin-A-treated mice. The significance between infected control and treated groups was calculated using one-way ANOVA with Dunnett’s posttest (*P < 0.05, **P < 0.01, and ***P < 0.001).

The disease outcome in the *Leishmania* infection is dependent on the cytokine environment (35). We investigated the mRNA expression levels of Th1 cytokines (IFN-γ and IL-12), Th2 cytokines (IL-10, IL-4, and TGF-β), and iNOS from spleen tissue of infected and treated mice using RT-qPCR. The iNOS transcript, a key factor in controlling the leishmaniasis, was significantly upregulated only with F6 (50 mg/kg b.wt.)- and withaferin-A-treated mice. However, we could not observe any significant alterations in the levels of proinflammatory cytokine IFN-γ in treated and infected control mice. The IL-12p40 expression was significantly increased in higher doses of F5-, F6-, and withaferin-A-treated mice compared to the infected control. IL-10, a key Th2 cytokine is important for the parasite persistence, was drastically reduced in F5-treated mice (50 mg/kg b.wt.) (*P* < 0.001), F6-treated mice (25 and 50 mg/kg b.wt.) (*P* < 0.01 and *P* < 0.001), and withaferin-A (*P* < 0.01)-treated mice when compared with infected control. Other Th2 cytokines IL-4 (*P* < 0.01) and TGF-β (*P* < 0.001) were also decreased in treated groups compared to the infected group (Figures 5A,B). The ratio of IFN-γ/IL-10 expression also signifies the extent of cure in F5-, F6-, and withaferin-A-treated groups compared to infected group (Figure 5C). The results indicate that withanolides suppresses the Th2-specific cytokines (IL-10, IL-4, and TGF-β) mRNA expression and promotes Th1-dependent parasite clearance in VL spleen.

### Withanolides Induces IgG2a Response

**Figure 6:**
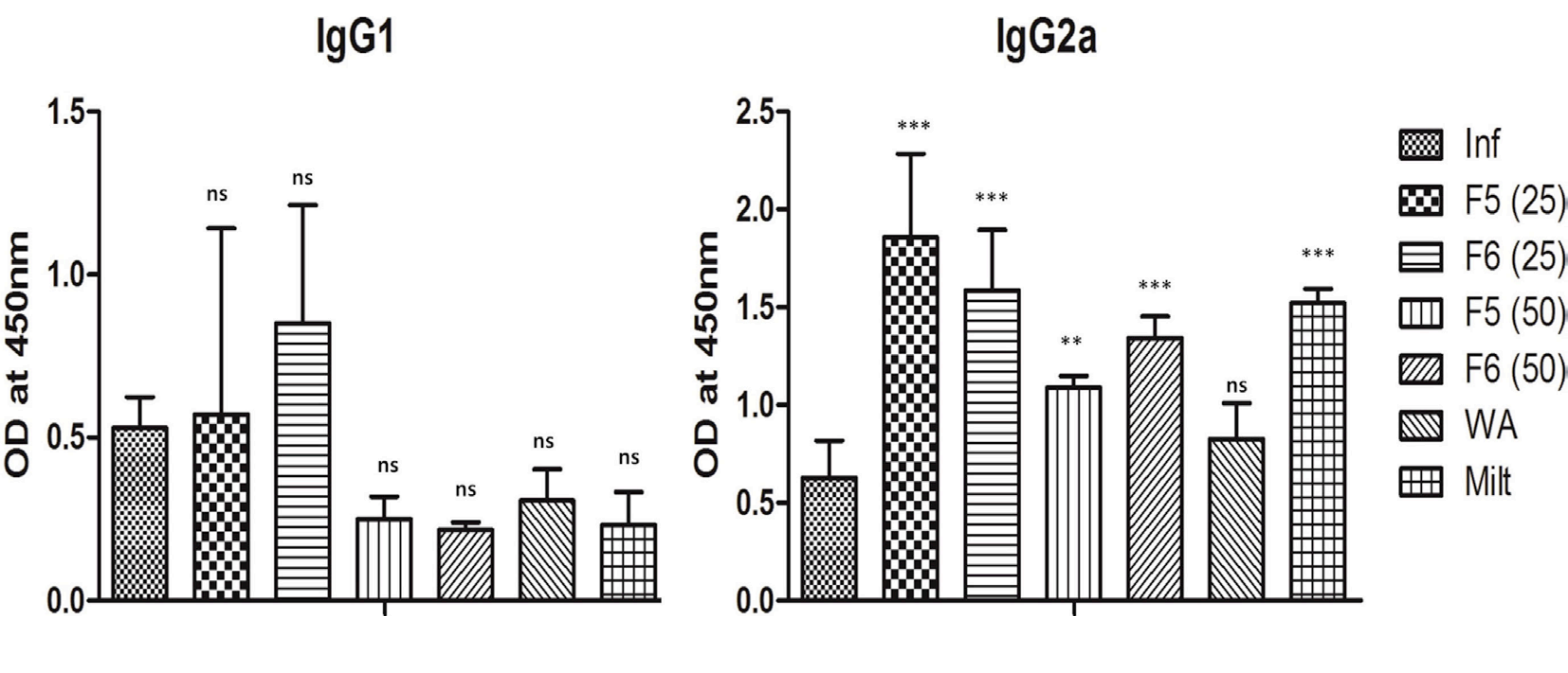
**The IgG1 and IgG2a antibody titer levels in different treatment groups of mice**. The results are expressed as mean ± SD of five mice. The significance was calculated between infected control and treated mice groups using one-way ANOVA with Dunnett’s posttest (*P < 0.05, **P < 0.01, and ***P < 0.001).

The immune activation during the leishmaniasis determines the outcome of the disease and the humoral response in the form of antibody production, which plays an important role. It is a known fact that IFN-γ and IL-4 direct the immunoglobulin class switching of IgG2a and IgG1, respectively. We estimated the amounts of abovementioned immunoglobulin present in the mice sera from all groups. We found a significant increase in IgG2a in F5 (*P* < 0.01), F6 (*P* < 0.001), a key marker of Th1 polarization in all the treated groups compared to control except in withaferin-A-treated groups. The decrease in IgG1 in the treated group was insignificant when compared with the infected group (Figure 6). The results indicate that withanolides induces the Th1 type of immune response in VL mice.

### Withanolides Elicits Granulomas Formation in Liver

**Figure 7:**
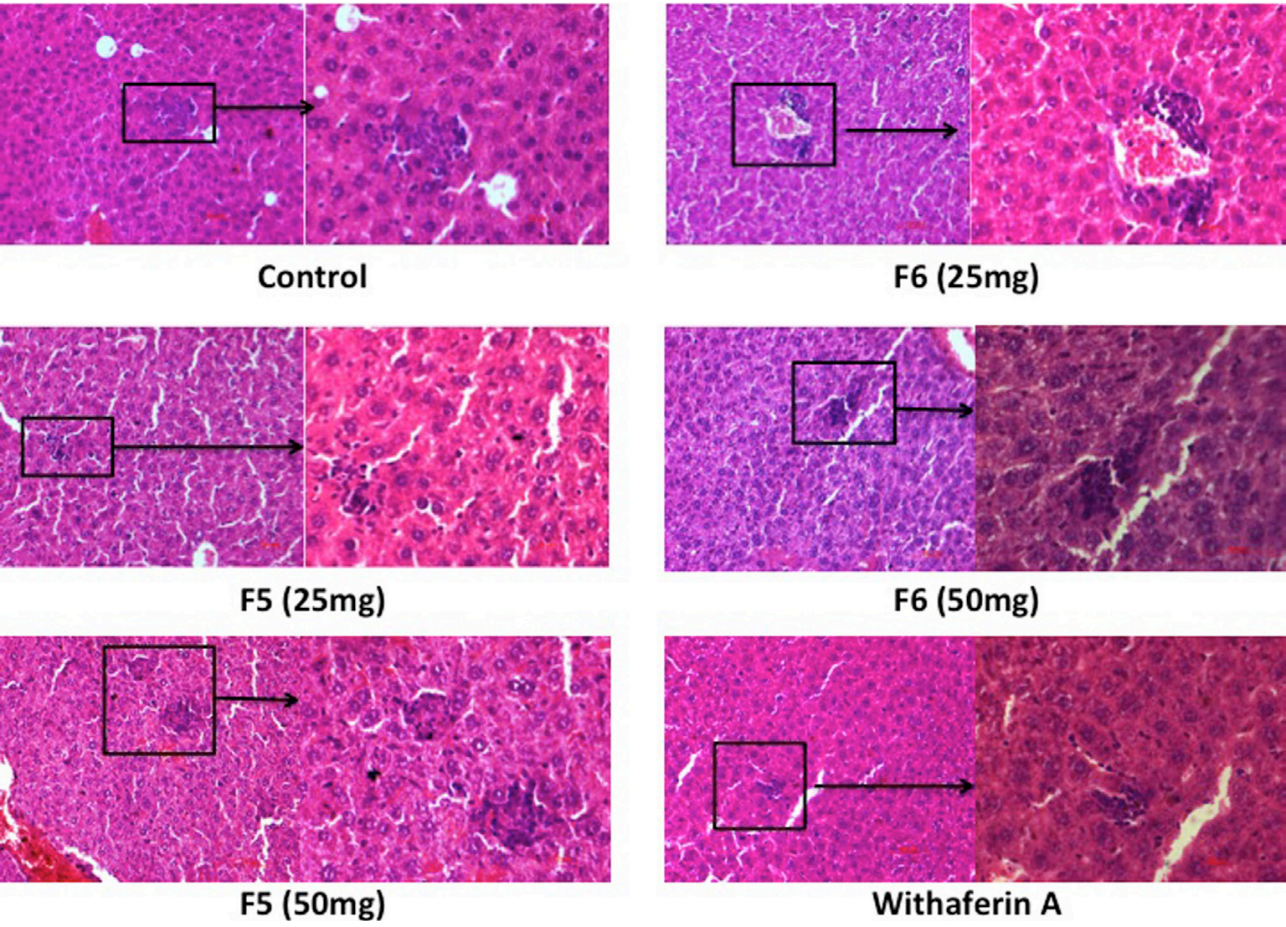
**Effect of F5, F6, and withaferin-A on histological changes in liver and hematoxylin and eosin-stained liver sections of different treatment mice groups**.

The liver section of the infected mice showed fatty degeneration of the tissue, which was restored in F5-, F6-, and withaferin-A-treated mice liver. During *L. donovani* infection, T lymphocytes get infiltrated in liver and develop into granulomatous structures harboring the amastigotes, thereby the infection is cleared in the liver with time. With F5 (25 mg/kg b.wt.) treatment, mice exhibited the formation of very loose and scattered granuloma. In F6 (25 and 50 mg/kg b.wt.) treatment, the granuloma structures were dense, tight, and more in number compared to the infected livers. Withaferin-A treatment also shows the same architecture when compared with other control (Figure 7). The result indicates that withanolides elicit the active granulomas formation in liver to clear the intracellular parasites.

## Discussion

The emergence of resistance to the existing drugs against leishmaniasis and HIV coinfection has been posing a significant challenge for the current chemotherapy (36). Plant-based products make an excellent repertoire for developing bioactive compounds against leishmaniasis (37). There are several reports in the past decade in the field, suggesting that plant products were used as immunomodulators in various infectious diseases (38, 39). The immune status of a host infected with *Trypanosoma* or *Leishmania* parasites played a significant role in successful chemotherapy. The treatment of African trypanosomiasis with melarsoprol requires an additional drugs suramin to enhance the efficacy of drug through antibody-mediated immune response. The treatment of VL with pentavalent antimonials together with macrophage activators has lowered the dose of antimony required to cure the infection, suggesting that therapeutic efficacy of antiparasitic drugs works in synchronization with the immune system (40). In VL, the immune system is severely compromised with leading to secondary infections and other complications associated with VL. The demand for antileishmanial drugs, which can effectively reverse the immune suppression besides being the leishmanicidal activity, needs to address on urgent basis.

*Withania somnifera* in the Indian traditional medicine system is considered as a wonder drug, which has many properties ranging from antibacterial to anticancer (41, 42). In our previous studies, we have shown that withanolides from *W. somnifera* leaves were able to induce apoptosis-like death in promastigote stage of *L. donovani* parasite (25). Hence, we want to further explore the antileishmanial activities of these withanolides in different concentrations of F5, F6, and withaferin-A on amastigotes stage of the parasite in the peritoneal macrophages and *in vivo* model of infection. We observed that more than 50% decrease in the parasite load in all treated macrophages compared to control. Our result was replicating the previous report, in which purified A6 fraction isolated from *W. somnifera* showed an IC_50_ value of 9.5 µg/mL in the amastigotes stage of the parasite (21). In the *in vivo* set up, there was a drastic reduction in parasite burden in spleen and liver with F5 (50 mg/kg b.wt.) and F6 (25 and 50 mg/kg b.wt.) but no change in the case of F5 (25 mg/kg b.wt.). However, F5 concentration (25 mg/kg b.wt.) showed a significant reduction in the hepatic parasite burden, but its futile action in spleen could be attributed due to the ineffective activation of Th1 cytokines in the spleen. With withaferin-A (2 mg/kg b.wt.) treatment, the reduction in spleen parasite burden was only 32%, which is comparatively less than that of F5 and F6 treatments. Recently, other group has shown prophylactic role of *W. somnifera* extracts in experimental VL and demonstrated that *W. somnifera* extract reduced the rate of infection in mice by increasing immunomodulatory activities (43). *Withania somnifera* extracts were also proven to be effective against the other parasite infections (17, 18).

Macrophage activation results in the enhancement of microbicidal activity through the upregulation of ROS and NO in the phagolysosomes, which is the key mechanism to control the *Leishmania* infection in the host (44, 45). Our results suggest that F5, F6, and withaferin-A might exert their antileishmanial activity via ROS production in peritoneal macrophages compared to their effect on infected PMM as it is one of the mechanisms used by the host in the absence of NO production. The lack of NO production by these withanolide fractions could be due to the unaltered IFN-γ expression both at mRNA and protein levels, as IFN-γ is an important mediator of NO production for the parasite killing in *Leishmania* infections (46, 47). However, we observed that the iNOS transcripts were significantly high in fraction F6, and withaferin-A could have activated the macrophage along with IL-12 cytokine for efficient killing of the parasite in phagolysosomes through activation of iNOS (48, 49). Repressions in cell-mediated immunity (CMI) and B-cell activation are the consequences of VL (50). CMI is important in mediating an effective Th1 type of immune response, which is important in controlling the *Leishmania* infection (51). During the *Leishmania* infection, an increased expression of IL-10 cytokine has been attributed to its ability to render macrophages deactivated, thereby nullify its microbicidal mechanism that exacerbates the severity of VL (52–54) and their neutralization promotes parasite clearance in VL spleen (55). Treatment of infected peritoneal macrophages with F5, F6, and withaferin-A demonstrated the significant decrease in IL-10 mRNA expression, which might contribute to the reduction in parasite load in PMM. However, the antigen-specific IFN-γ production in VL patients is not a limiting factor for their non-curative response (56, 57). The drastic increase in IFN-γ/IL-10 ratio in all the treated groups indicates the parasite clearance from the macrophages (58). The increased TNF-α levels in the culture supernatants of F6-treated PMM could be due to the increased ROS levels. Further studies are required to prove ROS as major driver of resistance against parasites. In our studies, we observed that F5, F6, and withaferin-A indeed upregulate mRNA expression of Th1 cytokines (IFN-γ and IL-12) and downregulate Th2 cytokines (IL-10, IL-4, and TGF-β), directing the immune response toward Th1 type with an increase in IFN-γ/IL-10 ratio, which supports the antileishmanial activity of withanolides.

*Leishmania* infection induces host IgG1 antibody response, which leads to an increased IL-10 secretion, promoting disease exacerbation (59). We found that Th1-specific IgG2a levels were increased in the treated mice compared to untreated mice, while there was no significant change observed in Th2-specific IgG1 levels. The higher IgG2a antibody titers in the treatments might be due to the increased IFN-γ production during the acute phase of VL. This indicates that withanolides from *W. somnifera* were possibly clearing the parasites from the host cell by precisely augmenting Th1 type of immune response. Histopathological changes in liver tissues reflect the successful clearance of parasites. During the hepatic immune response against the parasite, there is infiltration of immune cells at the infection site, followed by formation of the granulomas structure. With time, these granuloma structures clear the parasite and resolve itself (60). In the liver of infected mice, a huge infiltration of monocytes surrounding the parasites was observed, and the size of granuloma structures was found to be decreased with the treatment of F5, F6, and withaferin-A.

## Conclusion

Our study demonstrates that F5, F6, and withaferin-A had two different actions against *L. donovani* infection. First, they could induce the antileishmanial effect in both *ex vivo* and *in vivo* model, and second, they were able to direct the immune system toward the protective Th1 arm by suppressing the susceptible Th2 arm of the immune system. The study of other active principles, their mechanisms, and effect of these withanolides on various immune cells is in progress in our laboratory. Further studies will be required to confirm whether these withanolides can be used as a mixture of compounds or as single entities for the active antileishmanial and immunomodulatory activities.

## Ethics Statement

The 6- to 8-week-old female BALB/c mice were obtained from National Centre for Laboratory Animal Sciences (NCLAS), Hyderabad, India. The animals were kept and fed with water and mouse feed *ad libitum* in the Animal House facility of University of Hyderabad. All animal experiments were approved by the Institutional Animal Ethics Committee, University of Hyderabad (UH/IAEC/RSM/2014-I/29a).

## Author Contributions

RM and SS conceived and coordinated the study. SC and RM designed the experiments. SC and JV performed the experiments. SC, JV, and RM analyzed the experiments and contributed to the preparation of the figures. SC, JV, SS, and RM wrote the paper. All authors reviewed the results and approved the final version of the manuscript.

## Conflict of Interest Statement

The authors declare that the research was conducted in the absence of any commercial or financial relationships that could be construed as a potential conflict of interest.
